# Changes in Healthcare Professionals’ Practice Behaviors Through an Educational Intervention Targeting Weight Bias

**DOI:** 10.1007/s11606-024-09212-9

**Published:** 2024-12-18

**Authors:** Amanda Velazquez, Karen J. Coleman, Robert F. Kushner, Joseph F. Nadglowski, Patricia M. Nece, Jing Zhang, A. Janet Tomiyama

**Affiliations:** 1 Center for Weight Management and Metabolic Health, Jim and Eleanor Randall Department of Surgery, Cedars Sinai Medical Center, Los Angeles, CA USA; 2 Department of Research and Evaluation, Kaiser Permanente Southern California, Pasadena, CA USA; 3 Department of Health Systems Science, Kaiser Permanente Bernard J. Tyson School of Medicine, Pasadena, CA USA; 4 Department of Medicine, Northwestern University Feinberg School of Medicine, Chicago, IL USA; 5 Obesity Action Coalition, Tampa, FL USA; 6 Department of Psychology, University of California, Los Angeles, Los Angeles, CA USA

**Keywords:** weight bias, weight stigma, intervention, obesity, continuing medical education

## Abstract

**Objective:**

Weight bias is pervasive in healthcare and leads to worse patient outcomes. A uniquely designed 4-h continuing medical education (CME) intervention was assessed for changing healthcare professionals’ (HCPs’) weight biases and clinical practice behaviors.

**Design:**

The intervention used a (1) pre/post design examining CME attendees’ self-reported weight bias at baseline, after, and 4- and 12-month follow-up, and (2) post/post design examining obesity practice behaviors 12 months after intervention in attendees and non-attendees.

**Setting:**

Single medical center service area within Kaiser Permanente Southern California.

**Participants:**

All HCPs (*n* = 472) from the target service area were eligible to attend. Analyses were done with 218 HCPs who attended and 89 who did not.

**Methods and Analysis:**

The intervention contained theory-based elements of changing attributions of responsibility of obesity, increasing empathy, creating self-awareness of weight bias, and creating a bias-free culture*.* For pre/post analyses, the primary outcome was self-reported weight bias. For comparative analyses of CME attendees and non-attendees, the outcomes were electronic medical record–confirmed rates of obesity diagnosis and referrals to evidence-based obesity treatments in the 12 months following the CME intervention.

**Results:**

Self-reported negative obesity stereotypes were significantly reduced compared to baseline while self-reported empathy and confidence in caring for patients with obesity were significantly increased immediately post intervention and were maintained at 4- and 12-month follow-up. After adjusting for years in practice, race/ethnicity, gender, profession type, practice type, and panel size, HCPs who attended the CME intervention had significantly increased odds (range 60–212%) of diagnosis and obesity-related referrals in the 12 months following the CME intervention when compared to HCPs who did not attend.

**Conclusion and relevance:**

This intervention has promise to be a scalable program that goes beyond impacting HCP’s self-reported weight bias and also changes HCPs’ clinical practice behaviors related to obesity treatment.

**Supplementary Information:**

The online version contains supplementary material available at 10.1007/s11606-024-09212-9.

## INTRODUCTION

### Problem Description

Over 40% of adults in the United States (U.S.) have obesity defined as a body mass index (BMI) of 30 kg/m^2^ or more.^1^ The high prevalence of obesity may lead to more people experiencing weight bias, which can include discrimination, negative attitudes, and prejudice against those perceived to have higher weight.^1^ Indeed, data from a U.S. census-matched sample showed that 40% of individuals experience some form of weight bias.^2^ One context in which weight bias is particularly troubling is in healthcare. Physicians report significant weight bias,^3–6^ which can lead to healthcare avoidance by patients,^7–9^ resulting in delayed diagnoses and poorer patient outcomes.^10^ Therefore, interventions to eliminate weight bias are critically needed.

### Available Knowledge

Despite the importance of eradicating weight bias,^11^ the existing literature shows little evidence that weight bias interventions are effective.^12,13^ A systematic review of weight bias interventions in the healthcare setting^14^ identified four theory-based intervention strategies: (1) changing attributions of responsibility of obesity by highlighting biological and structural factors that determine body weight; (2) increasing empathy for those with obesity; (3) creating self-awareness of bias; and (4) using leadership to create a culture in which weight bias is discouraged. This review also highlighted the suboptimal methodology that had been used in studies testing these intervention strategies, further hindering progress in this area. The vast majority of the studies engaged undergraduate or medical students as participants, with only two studies engaging current practitioners.^14^ Most studies used pre-post designs with no comparison groups. The interventions tested often only used one of the four strategies, whereas the authors of the systematic review recommended that combining them is likely necessary to effect significant change. Moreover, most studies had short follow-up periods. Most importantly, none of the studies investigated actual practice changes, relying solely on physician attitudes as outcome measures.

### Rationale and Specific Aims

The current study was designed to address several of the methodological weaknesses of the existing literature^14^ and included (1) targeting practicing HCPs across the entire system rather than undergraduate and medical students, (2) comparing intervention participants to a sample of non-attendees, (3) including all intervention strategies identified in the systematic review,^15^ (4) including longer-term follow-up points at 4- and 12-months post-intervention, and (5) including objective behavioral practice differences among HCPs using electronic medical records (EMR). We hypothesized that this innovative, immersive obesity educational symposium would reduce weight bias among HCPs and result in clinical practice changes that increase access to obesity treatments for patients living with obesity.

## MATERIALS AND METHODS

### Context

A prospective interventional study was conducted among HCPs in one of 15 Kaiser Permanente (KP) medical center service regions located in Southern California.

### Participants

Participants eligible for the intervention were all HCPs who worked within the medical center service region that was the target of the CME intervention (*n* = 472). Details of recruitment are provided later in the description of the intervention. Of the 472 eligible HCPs, *n* = 250 attended the CME intervention. The remaining *n* = 112 did not attend. Further exclusions were made for analyses to exclude HCPs who did not have a scope of practice requiring obesity care such as inpatient, pediatrics (as the CME was about adult obesity), radiology, administration, and surgery (*n* = 23 excluded who did not attend; *n* = 32 excluded who did attend). This left a final sample for analyses of *n* = 218 HCPs who attended the CME intervention and *n* = 89 who did not.

### Patient and Public Involvement Statement

Patients were involved in both the design and conduct of the study. For 2 years prior to the study initiation, the principal investigator of the study (AV) heard from patients in clinical practice of the recurring barriers to accessing obesity treatments, with the most common barrier being HCPs’ biases barring access to referrals to medical weight management or bariatric surgery and medicine. As a result, patient experiences were a driving force for the design of the study (targeting education to HCPs on evidence-based treatment of obesity) and outcome measures (referral rates to various treatments for obesity). Patients were also involved in the conduct of the research. Specifically, KP patients delivered part of the intervention, in which they took part in a panel where they shared their experiences with weight bias with KP HCPs. Their lived experiences were designed to elicit empathy, and also served to increase awareness of weight bias as a salient problem at KP.

### Intervention

A core working committee of individuals with experience in obesity education was established in 2020 to develop the content for the symposium. This core committee worked closely with project managers, meeting planners, and KP patients living with obesity in the development of this program. Communication and planning were accomplished through virtual meetings, email exchanges, and conference calls.

Through an iterative process of approximately 1 year, the committee developed a program designed to be engaging, innovative, and personalized to KP HCPs (Supplementary Table S1). The agenda included various forms of education delivery, from patient panel discussions, faculty-led didactics, video viewing, question/answer periods, and simulated clinical scenarios. The key components of the intervention were as follows: (1) To change attributions of responsibility of obesity, didactic lectures were deployed to highlight uncontrollable biological and structural factors that determine weight; (2) To increase empathy, a panel of KP patients talked about their negative treatment experiences from HCPs; (3) To create self-awareness of weight bias, participants took an Implicit Attitudes Test^16^ that reported back results of whether they had pro-thin, anti-fat bias; and (4) To create a culture in which weight bias is discouraged, all participants were invited to sign a pledge to work on reducing weight bias and stigma of obesity in healthcare. The decision was made to deliver the CME intervention virtually, as a consequence of the COVID-19 pandemic.

Supplementary Table S2 displays the timeline of survey distributions, reminders, and marketing. Prior to the CME intervention, coordination with department chiefs was needed to ensure physicians’ scheduled educational time was designated to the CME symposium day of Thursday April 29, 2021. Participation was strongly encouraged by the Medical Director.

The 4-h CME symposium was delivered on April 29, 2021. To avoid significant disruption to patient access and safeguard a high level of participation, the intervention was repeated three times (AM, PM, and evening sessions). The content remained the same for all three sessions, of which 75% was pre-recorded to ensure consistency. During opening and closing remarks for each session, a link and QR code was made available to participants to access the pre- and post-survey. Participants were informed that CME credit could still be obtained if they chose not to participate in the pre/post and longitudinal survey assessments.

One month following the symposium, all attendees received a follow-up email sent by the Medical Director and the PIs of this study, which reviewed key takeaways and professional resources. Participants were emailed surveys for the 4- and 12-month follow-ups.

### Study of the Intervention

First, we used a longitudinal observational design to test pre/post changes (baseline vs. immediately post-intervention, 4 and 12 months) in weight bias for those who experienced the intervention. Second, we compared intervention participants to non-attendees to assess differences in practice behaviors between these two groups. This approach examined obesity diagnosis rates and referral rates to a healthy lifestyle program, obesity medicine, and bariatric surgery as the outcome measures. Practice behaviors of each physician were collected from the EMR regarding outpatient/inpatient/emergency/urgent care services. The index date was defined as the time of the symposium on April 29, 2021. As recommended when reporting system-level work to improve healthcare and evaluate the effect of interventions, we followed the Standards for Quality Improvement Reporting Excellence for Education (SQUIRE-EDU) guidelines.^15^

### Measures

#### Weight Bias

Weight bias was assessed using a 16-item questionnaire from Kushner and colleagues^17^ (see Supplementary Table S3) designed to assess negative attitudes and beliefs among clinicians toward patients with obesity. This measure captures three types of weight bias: negative obesity stereotypes (e.g., “Individuals with obesity have themselves to blame.”), empathy for patients with obesity (e.g., “People with obesity feel stigmatized by the medical profession.”), and confidence in clinical interaction with patients with obesity (e.g., “I feel comfortable talking to people about their weight.”). The original items were modified to reflect person-first language (e.g., “people with obesity” vs. “obese people”). Internal consistency for the *n* = 218 HCPs who attended the CME intervention for each type of weight bias were Negative Stereotypes Cronbach’s *α* = 0.66, Empathy *α* = 0.35, and Confidence *α* = 0.80.

#### EMR Data Abstraction

The primary outcomes of interest from the EMR were the practice changes of HCP symposium attendees of obesity diagnosis and referrals for healthy lifestyle programs, obesity medicine, and bariatric surgery. These outcomes were captured before and after the symposium for the 93,987 patients with obesity (BMI ≥ 30) with at least 1 visit. Covariate information was extracted from electronic sources and comprised physician demographics including age, race/ethnicity, gender, years in practice, practice type, professional type, and department.

### Analysis

Continuous data are reported as mean ± standard deviation (SD) and median ± the interquartile range (IQR). Unadjusted differences between time points are analyzed using paired sample *t*-tests and differences between groups with independent samples *t*-tests. Categorical data are presented as absolute values and percentages. Differences between time points are analyzed using nonparametric Fisher exact tests and differences between groups are analyzed using nonparametric chi-square tests.

To assess changes in weight bias for only those HCPs who attended the CME intervention and were eligible for the analyses (*n* = 218), analyses were conducted between baseline and post-intervention; post-intervention and 4 months; post-intervention and 12 months; and 4 months and 12 months. These paired comparisons were hypothesized *a priori* and thus significance levels were not adjusted for multiple comparisons. Listwise deletion was used.

The following four clinical practice behaviors were targeted as part of the CME intervention and abstracted from the EMR for all HCPs who were eligible for the analyses (*n* = 218 attendees and *n* = 89 non-attendees): (1) diagnosis of obesity for patients with obesity (BMI ≥ 30 kg/m^2^), (2) referral of patients with obesity to healthy lifestyle programs, (3) referral of patients with obesity to obesity medicine, and (4) referral of patients with severe obesity (BMI ≥ 35 kg/m^2^) to bariatric surgery. Univariate analyses were conducted for the 12 months before and 12 months following the CME intervention within each group of HCPs (pre/post for those who attended and pre/post for those who did not attend) and comparing the two groups of HCPs for the 12 months following the CME intervention (post/post).

Logistic regression models were utilized to derive the adjusted odds ratios (ORs) with 95% confidence intervals (CIs) to examine the four target practice behavior differences comparing the two groups of HCPs for the 12 months following the CME intervention. To control for confounding, all models were adjusted for years in practice, race/ethnicity, gender, professional type, practice type, and panel size. All analyses were two-sided and performed using SAS version 9.4 (Cary, NC). *P* values < 0.05 were considered statistically significant.

### Ethical Considerations

As the intervention was a CME program and did not contain sensitive data, the Institutional Review Board certified it exempt. IRB approval was obtained in April 2022 to abstract data from the EMR to assess practice changes.

## RESULTS

Table 1 displays demographic and practice characteristics of the sample. Physicians from Family Medicine, Internal Medicine, and Obstetrics-Gynecology were more likely to attend the CME symposium, while physicians from Orthopedics and Ophthalmology were less likely to attend (*p* < 0.001). The only other significant difference between those who did and did not attend was professional type, as physicians were more likely to attend the CME symposium than other types of professionals such as nurses (*p* = 0.003).

**Table 1:** Demographics of Healthcare Professionals Who Attended (*n* = 218) and Those Who Did Not Attend (*n* = 89) the Intervention

	Attendees (*N* = 218)	Non-attendees (*N* = 89)	Total (*N* = 307)	*p*
Age				.96*
Mean (SD)	45.9 (8.8)	45.8 (8.9)	45.9 (8.8)	
Median (IQR)	45.0 (39.0, 52.0)	46.0 (38.0, 53.0)	45.0 (39.0, 52.0)	
Age range, *n* (%)				.17^†^
28–39	55 (25.2%)	27 (30.3%)	82 (26.7%)	
40–45	65 (29.8%)	16 (18.0%)	81 (26.4%)	
46–52	50 (22.9%)	21 (23.6%)	71 (23.1%)	
52 +	48 (22.0%)	25 (28.1%)	73 (23.8%)	
Race/Ethnicity, *n* (%)				.57^‡^
Asian/Pacific Islander	89 (40.8%)	39 (43.8%)	128 (41.7%)	
White	89 (40.8%)	29 (32.6%)	118 (38.4%)	
Black	23 (10.6%)	14 (15.7%)	37 (12.1%)	
Hispanic	14 (6.4%)	6 (6.7%)	20 (6.5%)	
Other/Unknown	3 (1.4%)	1 (1.1%)	4 (1.3%)	
Gender, *n* (%)				.13^†^
Female	126 (57.8%)	43 (48.3%)	169 (55.0%)	
Male	92 (42.2%)	46 (51.7%)	138 (45.0%)	
Years in practice				.29*
Mean (SD)	11.5 (9.07)	12.4 (8.67)	11.8 (8.95)	
Median (IQR)	10.0 (4.0, 17.0)	11.0 (5.0, 20.0)	10.0 (5.0, 18.0)	
Practice type, *n* (%)				.09^†^
Adult outpatient	86 (39.4%)	26 (29.2%)	112 (36.5%)	
Adult specialty	132 (60.6%)	63 (70.8%)	195 (63.5%)	
Professional type, *n* (%)				.003^†^
M.D./D.O./DPM	195 (89.5%)	68 (76.4%)	263 (85.7%)	
Other	23 (10.5%)	21 (23.6%)	50 (14.3%)	
Average number of patients per professional				
Mean (SD)	1900 (1030)	1,6973 (957)	1842 (1012)	.15*
Median (IQR)	1854 (1070, 2574)	1482 (906, 2430)	1802 (984, 2479)	
Panel sizes, *n* (%)				.12^†^
1–999	48 (22.0%)	29 (32.6%)	77 (25.1%)	
1000–1999	77 (35.3%)	24 (27.0%)	101 (32.9%)	
2000 +	93 (42.7%)	36 (40.4%)	129 (42.0%)	
Department, *n* (%)				0.0001^‡^
Addiction Medicine	0 (0.0%)	1 (1.1%)	1 (0.3%)	
Allergy	2 (0.9%)	0 (0.0%)	2 (0.7%)	
Cardiology	5 (2.3%)	5 (5.6%)	10 (3.3%)	
Dermatology	7 (3.2%)	1 (1.1%)	8 (2.6%)	
Ear, Nose, and Throat	1 (0.5%)	0 (0.0%)	1 (0.3%)	
Endocrinology	3 (1.4%)	1 (1.1%)	4 (1.3%)	
Family Medicine	54 (24.8%)	18 (20.2%)	72 (23.5%)	
Gastroenterology	4 (1.8%)	4 (4.5%)	8 (2.6%)	
Geriatric Medicine	5 (2.3%)	3 (3.4%)	8 (2.6%)	
Head and Neck	4 (1.8%)	1 (1.1%)	5 (1.6%)	
Infectious Diseases	4 (1.8%)	0 (0.0%)	4 (1.3%)	
Internal Medicine	32 (14.7%)	8 (9.0%)	40 (13.0%)	
Psychiatry	14 (6.4%)	3 (3.4%)	17 (5.5%)	
Nephrology	5 (2.3%)	3 (3.4%)	8 (2.6%)	
Neurology	6 (2.8%)	1 (1.1%)	7 (2.2%)	
Obstetrics/Gyn	23 (10.6%)	8 (9.0%)	31 (10.1%)	
Occupational Health	5 (2.3%)	1 (1.1%)	6 (2.0%)	
Oncology	4 (1.8%)	2 (2.2%)	6 (2.0%)	
Ophthalmology	0 (0.0%)	9 (10.1%)	9 (2.9%)	
Orthopedics	3 (1.4%)	11 (12.4%)	14 (4.6%)	
Plastic Surgery	10 (4.6%)	4 (4.5%)	14 (4.6%)	
Pain Management	1 (0.5%)	2 (2.2%)	3 (1.0%)	
Physical Medicine	5 (2.3%)	1 (1.1%)	6 (2.0%)	
Podiatry	6 (2.8%)	0 (0.0%)	6 (2.0%)	
Pulmonology	5 (2.3%)	2 (2.2%)	7 (2.3%)	
Rheumatology	3 (1.4%)	0 (0.0%)	3 (1.0%)	
Urology	7 (3.2%)	0 (0.0%)	7 (2.3%)	

^*^Kruskal–Wallis *p*-value; ^†^chi-square *p*-value; ^‡^Fisher’s exact *p*-value

### Weight Bias Changes

Table 2 provides descriptive statistics and comparative findings for the three weight bias outcomes. Negative obesity stereotypes were significantly reduced compared to baseline (2.81 ± 0.47 vs. 2.50 ± 0.46; *t*(140) = 9.03; *p* < 0.001), while empathy (3.33 ± 0.64 vs. 3.47 ± 0.63; *t*(140) = − 2.77; *p* = 0.006) and confidence (3.10 ± 0.86 vs. 3.85 ± 0.79; *t*(140) = − 11.30; *p* < 0.001) were significantly increased. These changes were maintained over the 4- and 12-month time periods. Sample sizes at each time point reflected a proportion of baseline respondents (baseline *N* = 218, post-CME intervention *n* = 183 [84% of baseline sample], 4-month *n* = 79 [36%], and 12-month *n* = 44 [20%]). Given this high rate of dropout, we engaged in analyses comparing the baseline sample to the 12-month sample in terms of demographic characteristics, and they were not significantly different in years in practice (*Χ*^2^ = 1.22, *p* = 0.749), gender (*Χ*^2^ = 2.39, *p* = 0.122), race/ethnicity (*Χ*^2^ = 10.55, *p* = 0.103), or age (*Χ*^2^ = 2.91, *p* = 0.573).

**Table 2:** Results for Self-Reported Weight Bias for Patients with Obesity in Those Healthcare Professionals Who Attended the Continuing Medical Education Seminar (*n* = 218). Data Are Presented as Means ± Standard Deviation. Means Are Compared Using Paired-Sample *t*-Tests

	Baseline (*n* = 218)	Post (*n* = 183)	*p*	4-month follow-up (*n* = 79)	p*	12-month follow-up (*n* = 44)	p*	*p*^‡^
Negative stereotypes	2.81 ± 0.47	2.50 ± 0.46	< 0.001	2.53 ± 0.48	0.75	2.93 ± 1.35	0.08	0.21
Empathy	3.32 ± 0.64	3.47 ± 0.63	0.006	3.45 ± 0.68	0.06	3.48 ± 0.59	0.39	0.48
Confidence in treating	3.10 ± 0.86	3.85 ± 0.79	< 0.001	3.81 ± 0.79	0.66	3.75 ± 0.90	0.60	0.51
Overall score	2.97 ± 0.35	2.93 ± 0.33	0.45	2.94 ± 0.37	0.85	2.96 ± 0.38	0.76	0.39

^*^Compared to post; ^‡^comparing 12-month to 4-month

### Differences in Practice Behaviors

Table 3 summarizes the four clinical practice behaviors in the 12 months before the CME intervention and in the 12 months following the CME intervention by HCPs who did vs. did not attend the CME. For HCPs who attended the CME, all practice behaviors increased from 12 months before to 12 months after (*p* < 0.001); whereas for those HCPs who did not attend the CME symposium, their practice behaviors either decreased (*p* < 0.001; *p* = 0.02) or did not change (*p* = 0.75).

**Table 3:** Unadjusted Practice Behaviors for Those Healthcare Professionals Who Were Included in the Analyses (*n* = 307). Data Are Shown for the 12 Months Before the Continuing Medical Education Intervention and for 12 Months After the CME Intervention

	Workshop attendees (*n* = 218)			Non-attendees (*n* = 89)			All (*n* = 307)
	12 months pre-intervention	12 months post-intervention	*p* (pre vs. post)	12 months pre-intervention	12 months post-intervention	*p* (pre vs. post)	*p* (post vs. post)
Patients with obesity (BMI ≥ 30)	42,511	51,476	–-	22,993	26,388	–-	–-
Patients with obesity (BMI ≥ 30) with a diagnosis code of obesity, *n* (% yes)	10,996 (25.9%)	14,057 (27.3%)	< 0.001*	4858 (21.1%)	4704 (17.8%)	< 0.001*	< 0.001*
Patients with obesity (BMI ≥ 30) with a referral to healthy lifestyle programs, *n* (% yes)	2213 (5.2%)	3434 (6.7%)	< 0.001*	1428 (6.2%)	1325 (5.0%)	< 0.001*	< 0.001*
Patients with obesity (BMI ≥ 30) with a referral to obesity medicine clinic, *n* (% yes)	626 (1.5%)	1266 (2.5%)	< 0.001*	233 (1.0%)	275 (1.0%)	0.75*	< 0.001*
Patients with severe obesity (BMI ≥ 35) with a referral to bariatric surgery, *n* (% yes)	265 (1.3%)	513 (2.1%)	< 0.001*	121 (1.1%)	101 (0.8%)	0.02*	< 0.001*

Table 4 presents the results of the adjusted logistic regression model comparing intervention participants to non-attendees for each of the practice behaviors of interest in the 12 months following the CME seminar. After adjusting for years in practice, race, gender, professional type, practice type, and panel size, there were significant differences between those who did and did not attend the CME intervention in all practice behaviors. When compared to those HCPs who did not attend the CME intervention, those who did attend had a 60% increased odds for diagnosing obesity (OR = 1.60; 95% CI 1.54,1.66), 27% increased odds of referring to healthy lifestyle programs (OR = 1.27; 95% CI 1.19,1.36), 87% increased odds of referral to obesity medicine specialty clinic (OR = 1.87; 95% CI 1.63,2.14), and for those patients with a BMI ≥ 35 kg/m^2^, 2.12 times the odds of referral to bariatric surgery (OR = 2.12; 95% CI 1.70,2.67) in the 12 months following the CME intervention.

**Table 4:** Adjusted Differences in Practice Behaviors by Intervention Group for Those Healthcare Professionals Who Were Included in the Analyses (*n* = 307). Data Are Compared for Practices 12 Months Following the Continuing Medical Education Intervention. Data Were Analyzed Using Separate Logistic Regressions for Each Practice Behavior. Each Model Was Adjusted for Years in Practice, Race/Ethnicity, Gender, Professional Type, Practice Type, and Panel Size (see Table 1 for descriptive statistics for these covariates)

Practice behavior	OR	95% CI
Patients with obesity (BMI ≥ 30) with a diagnosis code of obesity		
Did not attend	Ref	–-
Attended	1.60	(1.54,1.66)
Patients with obesity (BMI ≥ 30) with a referral to healthy lifestyle programs		
Did not attend	Ref	–-
Attended	1.27	(1.19.1.36)
Patients with obesity (BMI ≥ 30) with a referral to obesity medicine clinic		
Did not attend	Ref	–-
Attended	1.87	(1.63,2.14)
Patients with severe obesity (BMI ≥ 35) with a referral to bariatric surgery		
Did not attend	Ref	–-
Attended	2.12	(1.70,2.67)

### Unintended Consequences

There were two unintended consequences of the intervention. First, during the development stages of the symposium, numerous KP non-physician HCPs (i.e., advanced practice professionals, midwives), expressed interest in participating in the symposium, and as a result, an invitation was extended to these HCP groups. Second, as a result of the intervention’s significant impact on practice changes leading to a twofold increase in referral patterns to the obesity medicine clinic, the BMI eligibility for new referrals was increased from BMI ≥ 30 to BMI ≥ 35 to address the overwhelming demand.

## DISCUSSION

Weight bias is pervasive in healthcare settings^6,14^ and may lead to worse patient outcomes.^10^ This intervention was aimed at reducing weight bias among HCPs and changing their obesity-related clinical practice behaviors. To our knowledge, it is the first of its kind to overcome the methodological weaknesses of the existing literature^14^ such as self-reported outcomes, and deliver an innovative CME intervention to a large cohort of mainly physician HCPs. Compared to 12 months prior to the CME intervention, in the 12 months following the intervention, there were 60% increased odds for diagnosing obesity, 27% increased odds of referring to healthy lifestyle programs, and 87% increased odds of referral to obesity medicine specialty clinic for those patients with a BMI > 30 kg/m^2^. The odds of a referral to bariatric surgery for patients with a BMI ≥ 35 kg/m^2^ increased by 2.12-fold in the same period. The comparison group’s odds either decreased or did not change.

The most notable strength of this study was the focus on objective changes in clinical practice behaviors as an outcome. The prior literature has focused on pre/post changes in practitioner attitudes without investigating whether actual practice behaviors shifted. Although we similarly observed self-reported changes similar to the prior literature^14,18^ in empathy, negative obesity stereotypes, and confidence in treating individuals with obesity, such measures are vulnerable to desirability bias, in which participants report attitudes that are in line with demand characteristics. Examining objective practitioner behavior, therefore, is important both from a basic scientific perspective and an applied perspective—without meaningful changes to actual practice, the time and financial costs of an intervention are wasted. Other key strengths and unique contributions to the literature included a comparison group that did not attend the CME intervention, the 1-year follow-up in self-reported attitudes and objective practice behaviors, and the comprehensive nature of the intervention that included the elements of changing attributions of responsibility of obesity, increasing empathy, creating self-awareness of weight bias, and creating a culture in which weight bias is discouraged.

There were also several limitations with the study that should be considered when interpreting the results. It was not possible to use randomization when delivering a CME intervention embedded in clinical practice and thus comparisons between those exposed to the intervention and those who were not should be treated with caution. We did attempt to adjust for confounders like practice type and years of service that might affect both participation in the CME intervention and practice change due to the CME intervention; however, there could be other variables that could explain our effects. In addition, only 20% of respondents completed the weight bias survey at 12 months and may not be reflective of the whole sample who attended the CME intervention. It is possible that those professionals who did not respond were those whose attitudes did not change. Also, the study was conducted at a single medical center service region in an integrated healthcare system, which limits generalizability to other settings with different healthcare models. However, the patient population of this center reflects significant racial and ethnic diversity, with Black patients making up the plurality of the EMR data, followed by Hispanic patients. Finally, the use of BMI as a measure has known limitations^19–22^ and the American Medical Association has issued a policy^23^ de-emphasizing BMI.

The implications of this study are numerous. Our data indicated that 3061 more patients (28% increase) received a diagnosis that could have led to services tied to obesity treatment; 1221 additional patients (55% increase) received a referral to a healthy lifestyle program; 640 additional patients (51% increase) received a referral to an obesity medicine specialty clinic; and 248 more patients (94% increase) received a referral to bariatric surgery. Offering the 4-h curriculum multiple times in a day avoided significant disruption to patient care. Moreover, the predominantly virtual nature of the intervention allows for scalable future dissemination. This intervention could be tailored for priority target populations, such as primary care physicians, who often serve as the gatekeepers to specialty care and obesity treatments.

Future research should focus on a greater integration of obesity pharmacotherapy treatment such as GLP-1 agonists into the CME content and an examination of practice behaviors related to medication management of obesity. In addition to a randomized trial of the intervention, future research should also assess longitudinal practice changes beyond 1 year. Through an innovative and focused CME intervention aimed at mitigating weight bias, HCPs’ behavior led to improved diagnosis and referral to obesity care for patients. This scalable, virtual obesity educational intervention has promise for changing HCPs’ clinical practice behaviors in other healthcare settings.

## Supplementary Information

Below is the link to the electronic supplementary material.

Supplementary file1 (DOCX 673 KB)

## Data Availability

The datasets generated during and/or analyzed during the current study are not publicly available due to the fact that they comprise patient medical records protected by HIPAA but are available from the lead site investigator on reasonable request. The unpublished data are only available for use through collaboration with the study investigators, a data use agreement upon which all parties must agree, and external funding. Persons interested in collaborating with the study team can contact Dr. Karen Coleman [Karen.J.Coleman@kp.org], the lead site investigator. We are eager to share this resource with others in collaboration to extend the evidence-base for effective obesity treatments.
