# Supplementary material for: Changes in Healthcare Professionals’ Practice Behaviors Through an Educational Intervention Targeting Weight Bias
Source: J Gen Intern Med. 2024 Dec 18;40(8):1720–7. doi: 10.1007/s11606-024-09212-9 (PMC12119418; doi:10.1007/s11606-024-09212-9)
Supplement: Supplementary file 1 — Supplementary file1 (DOCX 673 KB) [file 11606_2024_9212_MOESM1_ESM.docx]

**ONLINE SUPPLEMENTARY MATERIALS**

**Supplementary Table S1.** Continuing Medical Education (CME) intervention agenda


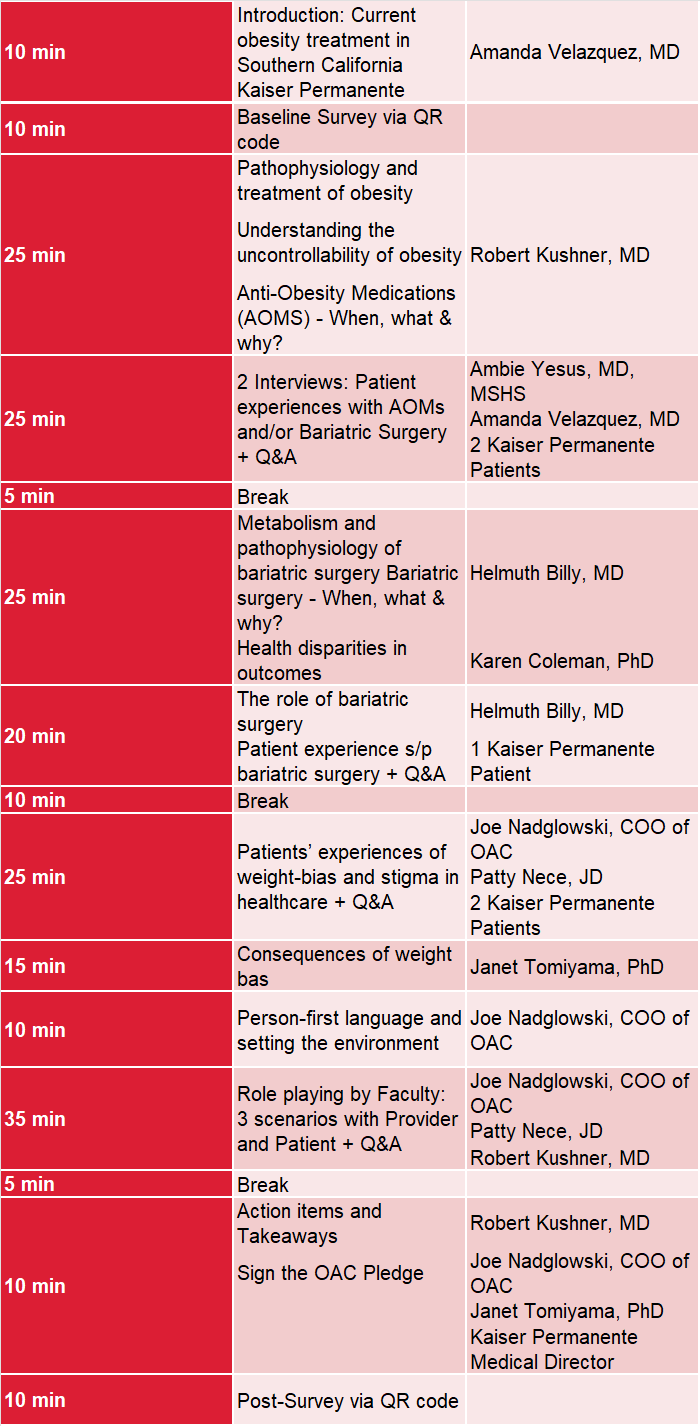


OAC = Obesity Action Coalition

**Supplementary Table S2.** Timeline of survey distribution, reminders and marketing


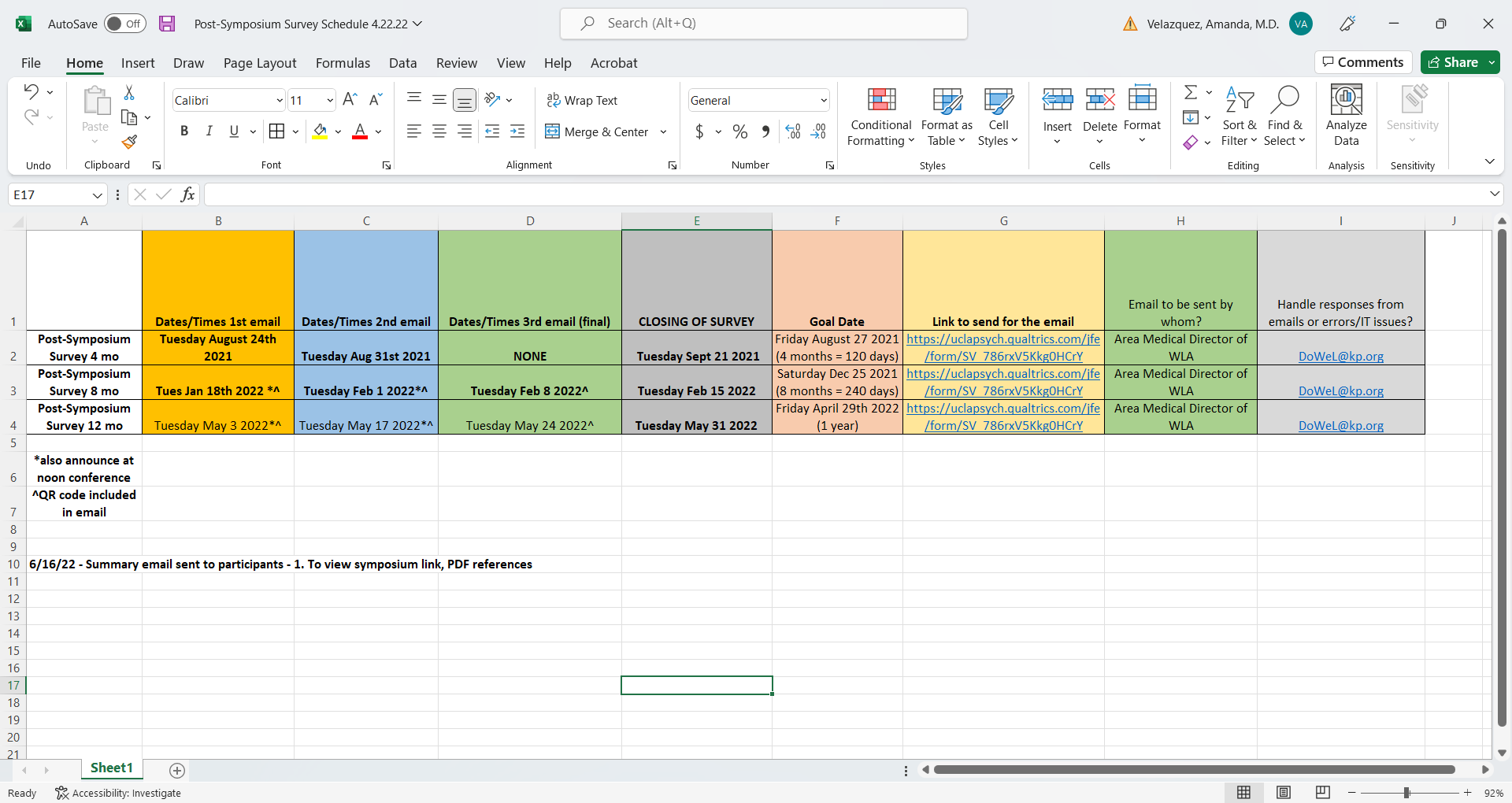


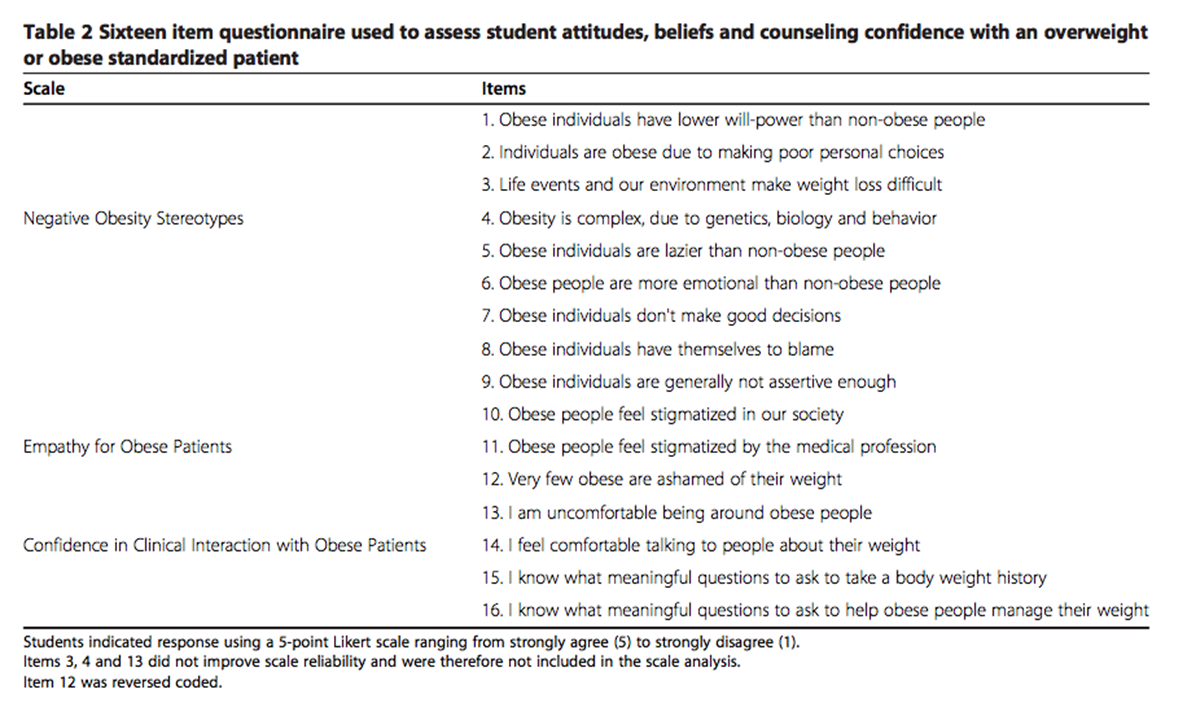
**Supplemental Table S3**. Questionnaire used to assess student attitudes, beliefs, and counseling confidence with a standardized patient with overweight or obesity. In the current study, we changed the language of the items to reflect person-first language (e.g., Item 1: “Individuals with obesity have lower will-power than people without obesity”). (Reprinted from Kushner et al., 2014)^1^

**ONLINE SUPPLEMENTARY REFERENCES**

1. Kushner RF, Zeiss DM, Feinglass JM, Yelen M. An obesity educational intervention for medical students addressing weight bias and communication skills using standardized patients. *BMC Med Educ*. 2014;14(1):53. doi:10.1186/1472-6920-14-53
